# Screening of unruptured intracranial aneurysms in 50 to 60-year-old female smokers: a pilot study

**DOI:** 10.1038/s41598-021-02963-z

**Published:** 2021-12-09

**Authors:** Justiina Huhtakangas, Jussi Numminen, Johanna Pekkola, Mika Niemelä, Miikka Korja

**Affiliations:** 1 Department of Neurosurgery, University of Helsinki and Helsinki University Hospital, P.O. Box 266, 00029 Helsinki, Finland; 2 Department of Radiology, University of Helsinki and Helsinki University Hospital, P.O. Box 266, 00029 Helsinki, Finland

**Keywords:** Neurology, Neurological disorders, Cerebrovascular disorders

## Abstract

The prevalence of unruptured intracranial aneurysms (UIAs) is around 2–3% in the general population. We hypothesized that the prevalence of small UIAs is higher among 50 to 60-year-old female smokers, since the incidence of aneurysmal subarachnoid hemorrhage (aSAH) is exceptionally high in 60 to 70-year-old female smokers. Ethics approval for this pilot study of 50 women was obtained from the hospital ethics committee. In order to minimize recruitment bias, preliminary invitation letters were sent to 50 to 60-year-old women who were known to be active smokers. Those interested in participating were further informed about the study rationale and protocol. Following written consent, participants filled a detailed questionnaire and underwent computed tomography angiography (CTA) analysis. All abnormalities were recorded. Of the 158 preliminary invitation letters, 70 potential participants initially replied. Of these, 50 returned questionnaires and written consents, 43 of which underwent CTA analysis. Most (39; 91%) were postmenopausal, and 9 (21%) were hypertensive. Two reported a family history (≥ 1 first-degree members) of intracranial aneurysms. UIAs (maximum sizes of 2, 2, 3, 3 and 7 mm) were found in five (12%) female smokers. One woman was operated on, and the remaining four were treated with non-invasive preventive actions (smoking cessation and follow-ups). Small UIAs, which may be best suited for non-invasive preventive actions, may be relatively common in 50 to 60-year-old female smokers. Whether this kind of targeted screening leads to improved health in female smokers requires further investigation.

## Introduction

The prevalence of unruptured intracranial aneurysms (UIAs) in the general population is poorly known. The population-based Rotterdam Study invited 45-year-old and older people from a suburb of Rotterdam to undergo standard (no angiographies) brain magnetic resonance imaging (MRI) screening. The results of this study indicated that UIAs were diagnosed in 35 of the 2000 (1.8%) study subjects (mean age 63.3 years; range 45.7–96.7)^1^. According to the pooled data of heterogenous studies reporting the prevalence of UIAs, around 3.2% of people with a mean age of 50 years (50% women) and without comorbidities carry UIAs^2^. A highly cited Finnish familial screening study found UIAs in 38 (8.7%) of the 438 asymptomatic first-degree family members (mean age 49 and 47 years in women and men, respectively; 53.2% women) from 85 families with two or more relatives with a possible history of intracranial aneurysms (IAs)^3^. According to the guideline published in 2015 for healthcare professionals, the American Heart Association/American Stroke Association deems UIA screening appropriate in families with two or more affected persons with IAs^4^.

Smoking is not only associated with, but also causes, SAH^5^. Moreover, SAH deaths are the most common type of stroke deaths in middle-aged women^6^. The incidence of SAH in women surpasses that of men at the age of 60 years^7^, and the incidence is highest among 70 to 75 -year-old women^7^. Sex and smoking habits affect the risk of SAH such that heavy female smokers have the highest risk of both SAH and sudden SAH death^8,9^. In fact, the only lifelong follow-up study thus far suggests that nearly 50% of female smokers with diagnosed UIAs suffer from SAH at some point in their lives^10^.

We hypothesized that risk factor-based targeted screening is capable of detecting a fair percentage of UIAs in a population with a high risk of SAH (not only a high risk of UIAs), and that this screening is safe, reasonable in cost, and widely available. Furthermore, we hypothesized that UIAs in this population are still small (< 5 mm), i.e. in a “preclinical state”, and therefore primarily suitable for non-invasive preventive actions such as smoking cessation and blood pressure lowering (if necessary). If true, the number of major complications related to invasive treatments of screen positives could be minimized. Based on these assumptions, we screened UIAs in 50 to 60-year-old female smokers using computed tomography angiography (CTA).

## Methods

### Ethical considerations

The Finnish Institute for Health and Welfare (THL) Biobank granted access to identify potential study participants from the previous studies coordinated by the THL Biobank. This pilot study of 50 participants (TYH2019107) was approved by the University Hospital Ethics Committee (HUS/1003/2019). Each participant signed a written informed consent before enrolment. The study was conducted in accordance with the principles of the Declaration of Helsinki^11^. Pseudonymized research data are personal data according to Finnish legislation and cannot be shared outside the Helsinki University Hospital.

### Recruitment protocol

**Figure 1:**
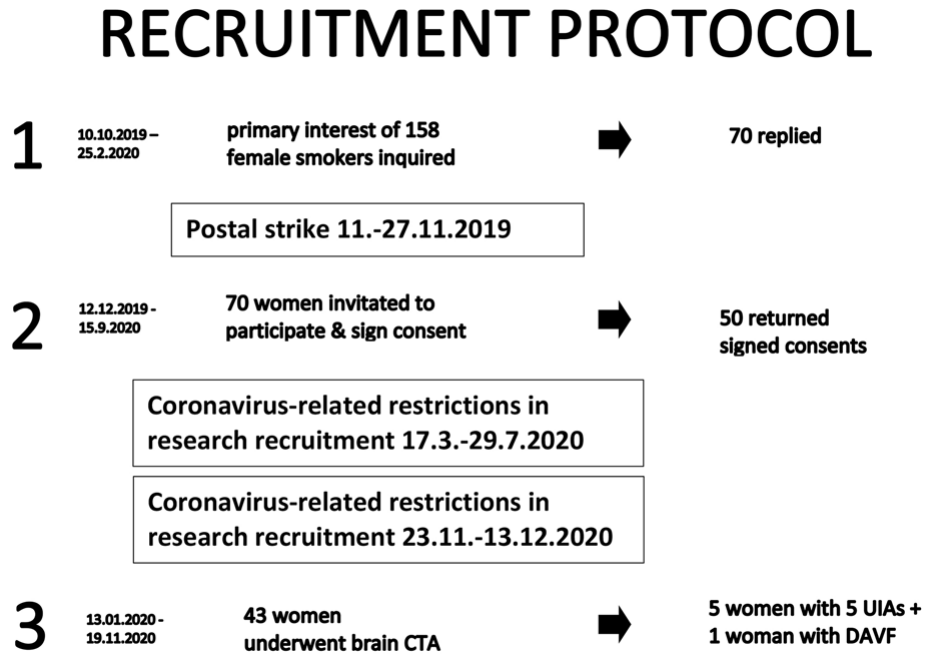
Schematic figure of the UIA screening -pilot study recruitment protocol.

In order to minimize recruitment bias, the Finnish Institute for Health and Welfare sent preliminary invitation letters to female smokers between the ages of 50 and 60 years old (age when study recruitment started; Fig. 1), who had previously participated in the GeneRISK study (https://thl.fi/en/web/thl-biobank/for-researchers/sample-collections/generisk-study). Briefly, the GeneRISK study is an ongoing prospective observational study focusing on genetic risk factors of cardiovascular diseases. In the GeneRISK study, 7342 randomly selected 45 to 65-year-old individuals were recruited between 2015 and 2017 in Southern Finland. Of these participants who had previously replied to a GeneRISK study questionnaire about smoking habits, current smokers were identified. The Finnish Institute for Health and Welfare posted preliminary invitation letters between October 2019 and February 2020 to potential participants. The preliminary invitation letter explained shortly the UIA screening study protocol and rationale. If a person was interested in the screening study and replied to the letter, the Finnish Institute for Health and Welfare securely shared their name and address with the study authors (JH and MK). Between December 2019 and September 2020, the study authors (JH and MK) and a research nurse sent the formal invitation letters (Fig. 1), which included a full description of the study protocol, instructions, contact details, questionnaires and consent, to the women who were preliminarily interested in participating. All invited participants who returned the signed consent were eligible to self-book a brain CTA examination appointment at the study hospital. Prior to the brain CTA examination, participants were also instructed to have a plasma creatinine test in any of the multiple University Hospital laboratories around Southern Finland. Participation in the study was not compensated, but documented travel expenses (use of own car or public transport) were reimbursed.

### Questionnaires

The study questionnaire, which was multiple choice and fill-in format, had 53 distinct questions divided into 7 domains: (1) health (2) smoking (e.g. smoking habits, regularity, daily amounts, smoking years, exposure to passive smoking and estimated ability to quit), (3) educational attainment and employment, (4) physical activity, (5) alcohol consumption, (6) hormonal status and (7) health-related measurements (height and weight).

### Quality of life

The participants were asked to complete two health-related quality of life (HRQOL) questionnaires (15D^12^ and EQ-5D-3L^13^), prior to and after the CTA screening, regardless of the CTA findings. For this work, we chose to use the 15D instrument, which has more dimensions than EQ-5D-3L^14^. The 15D is developed in Finland^15,16^ and is used as a generic and comprehensive instrument for measuring participants’ health-related quality of life in 15 dimensional measures (mobility, vision, hearing, breathing, sleeping, eating, speech, excretion, usual activity, mental function, discomfort/symptoms, depression, distress, vitality and sexual activity^17^). The 15D score is a sum (single number) of the 15 dimensions and is presented on a continuous scale between 0 and 1 (0 = dead, 1 = no problems in any dimensions). The generic minimum important change (MIC) for two longitudinal 15D scores is ± 0.015^18^. A change of > 0.035 is considered to be a large improvement or deterioration in the HRQOL^18^.

### CTA screening protocol

All CTA screens were performed in the Töölö hospital, where the Department of Neurosurgery is also located. The CTAs were acquired helically with a multi slice CT-scanner (GE Lightspeed VCT 64, GE Healthcare, USA) using the standard institutional protocol (120-kV tube potential, NI 10/100–500 mA). All CTA screens were performed with manual bolus tracking in the common carotid artery with a contrast material (350 mg I/mL) introduced at an injection rate of 5 mL/s, followed by 40 mL of saline. Axial raw data images were reconstructed with a slice thickness of 0.625 mm.

### Image analysis

In addition to the thin-slice raw data, 22/3 mm and 1 mm MIP axial, coronal and sagittal images were reconstructed and sent to the local PACS system, and then further to 3D workstation servers (syngo.via, Siemens Healthineers; or Vitrea, Vital, Canon Medical Systems) for 3D analysis by three observers (MK, JP, JN). Any disagreements between the observers (MK, JP, JN) were verified with digital subtraction angiography (DSA). Study participants with abnormal imaging findings were invited to the outpatient clinic, whereas participants without abnormalities were informed by mail.

## Results

### Recruitment protocol

The original GeneRISK study included 291 women born between the years 1958 and 1969 (study recruitment started in 2019), who were daily smokers, and who lived in Southern Finland. The study recruitment protocol is described in Fig. 1. In brief, between October 2019 and February 2020, the Finnish Institute for Health and Welfare sent preliminary invitation letters to 158 smoking women (Fig. 1), who lived within approximately 150 km of the study hospital. Of these 158 women, 70 expressed their preliminary interest to participate in the study (Fig. 1). At the end, 43 underwent a brain CTA screen (Fig. 1). The last brain CTA was done in November 2020 (Fig. 1). A postal strike in Finland and COVID-19 pandemic-related restrictions in the recruitment of research participants in the University Hospital complicated and extended the recruitment process (Fig. 1).

### Study cohort

Most of the participants were postmenopausal (n = 39, mean and median age 57 years), who considered that their own general health was very or fairly good (very good n = 3, fairly good n = 30, average n = 10). The most commonly reported long-term health concerns were hypercholesterolemia (n = 16), arthritis (n = 12), and hypertension (n = 9).

The participants had a history of 24 pack-years (mean and median pack-years 24 and 23) and 36 active smoking years (mean and median smoking time of 36 years). Two women (5%) reported that they quit smoking after receiving the formal invitation letter. Nine participants (21%) reported in the questionnaire that they could be able to quit smoking, but the majority were hesitant (n = 27, 63%); five participants (12%) estimated that they could not quit at all. Two (5%) of the 43 participants reported a family history (≥ 1 first-degree relatives) of UIAs.

### Screening results

**Figure 2:**
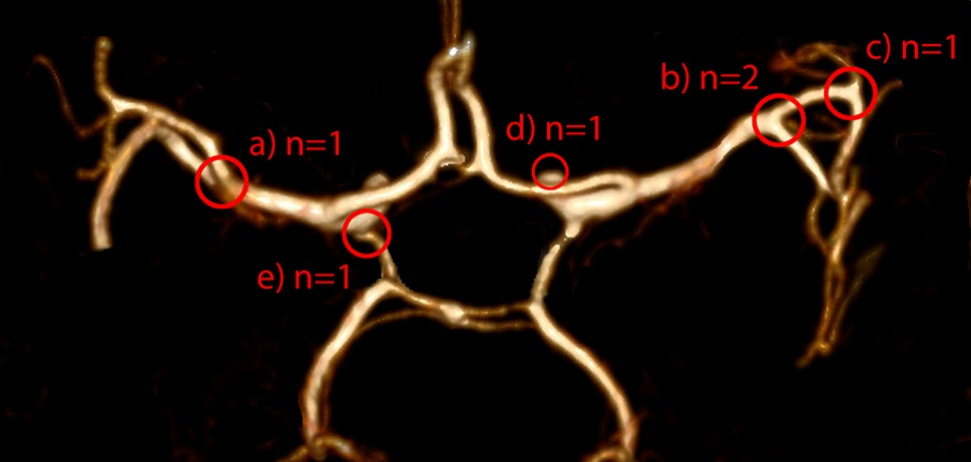
Locations of the six suspected UIAs along the circle of Willis detected in CTAs: (**a**) at the main bifurcation of left MCA, (**b**) at the main bifurcation of right MCA, (**c**) at the M2/3 bifurcation of the right MCA, (**d**) at the right ICA-ophthalmic segment, (**e**) at the left ICA- posterior communicating artery.

**Figure 3:**
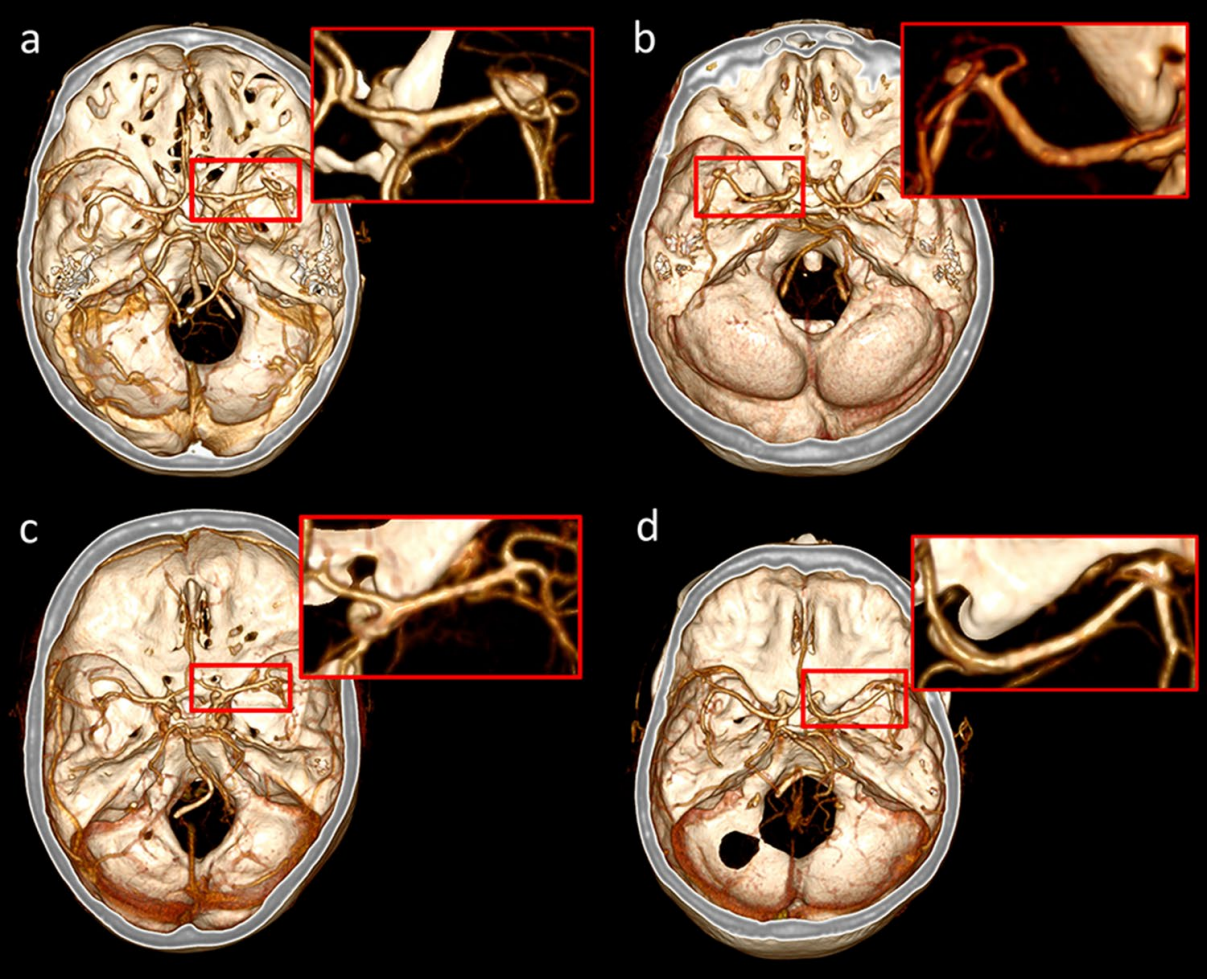
Four confirmed UIAs: (**a**) at the main bifurcation of right MCA (7 mm), (**b**) at the bifurcation of left MCA (3 mm), (**c**) at the M2/3 bifurcation of the right MCA (2 mm), (**d**) at the main bifurcation of right MCA (3 mm).

Screening results are reported in Table 1 and visualized in Fig. 2. None of the participants reported that they had previously undergone head CTA screens. However, electronic patient files and imaging archives revealed that one study participant had undergone a familial UIA screening in 2007. This participant did not have UIAs, neither in 2007 nor in our screening. A total of six suspected UIAs (maximum sizes of 2, 2, 2, 3, 3 and 7 mm) were identified in the CTAs of six (14%) patients (one per patient; Fig. 2), four of which (9%) were undisputable (Fig. 3a–d). The other two (2 mm and 2 mm in size) were not completely agreed upon by all authors, therefore the CTA findings were verified with DSA. One UIA suspicion was confirmed to be an infundibular dilatation of the posterior communicating artery, while the other could not be confirmed (Supplementary Figure) since the participant repeatedly cancelled the scheduled DSA. One participant had a dural arterio-venous fistula of the floor of the anterior cranial fossa.

**Table 1:** Screening results of 43 participants.

	CTA-positives (n = 5)	CTA-negatives (n = 38)	Total/All participants (n = 43)
Age at CTA (mean)	58	57	57
Diagnosed hypertension	2	7	9
**BP measured previously:**			
Last 6 months	4 (80%)	23 (61%)	27 (63%)
Within 6–12 months	1 (20%)	10 (26%)	11 (26%)
Within 1–5 yrs	–	5 (13%)	5 (12%)
UIA or SAH in family (first-degree relatives)	1	2	3
Number of suspected UIAs	5	1	6
Number of verified UIAs	4	0	4
UIA location			
MCA	4		4
ICA-opthalmic	1		1
ICA-other	0		0
AcomA or DACA	0		0
Posterior circulation	0		0
Other findings (other than UIA)	0	1	1
Need for DSA angiography	1	2	2
Complications, CTA or DSA	0	0	0
Active treatment for a pathology (neurosurgical or endovascular)	1	1	2
Conservative Treatment for a pathology (preventive actions and/or follow-up)	4	0	4

*CTA* Computed Tomography Angiography, *UIA* Unruptured Intracranial Aneurysm, *MCA* Middle Cerebral Artery, *ICA* Internal Carotid Artery, *AcomA* Anterior Communicating Artery, *DACA* distal anterior cerebral artery, *DSA* digital subtraction angiography or catheter angiography, *BP* blood pressure.

### Treatment

Smoking cessation and weekly blood pressure monitoring at home were strongly recommended for all patients. A 1-year follow-up CTA imaging study and a subsequent clinical visit was scheduled for the four patients with small (2–3 mm in size) UIAs. One patient with a 7 mm middle cerebral artery bifurcation aneurysm (Fig. 3a) was operated on. The surgery was uneventful, and the neurologically intact patient was discharged home two days after the surgery. She was on a standard sickness allowance for four postoperative weeks (20 working days). One patient with a dAVF underwent a DSA for treatment planning, and her treatment was scheduled for summer 2021.

### Quality of life

**Figure 4:**
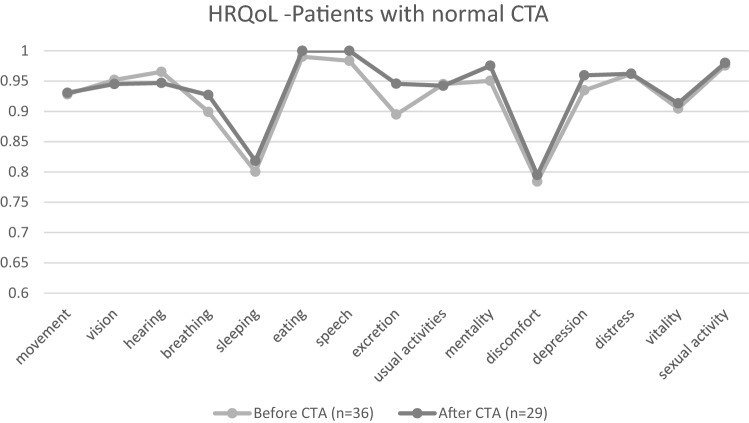
Health-related quality of life dimensions (15D score) of the participants prior to and after normal CTA results.

All participants filled the 15D at enrolment (before CTA imaging). Of the 43 participants, 34 also returned the 15D after receiving the CTA results, which revealed a mean increase of 0.010 (median 0.007) in the score. Specifically, 11 had the MIC (≥ 0.015) for better score, and 8 for worse. Four participants experienced large deterioration in the 15D score (> 0.035, one was CTA-positive), whereas 9 experienced large (> 0.035) improvement (none were CTA-positive). Figure 4 presents HRQoL-dimensions (15D) of patients with normal CTAs.

**Figure 5:**
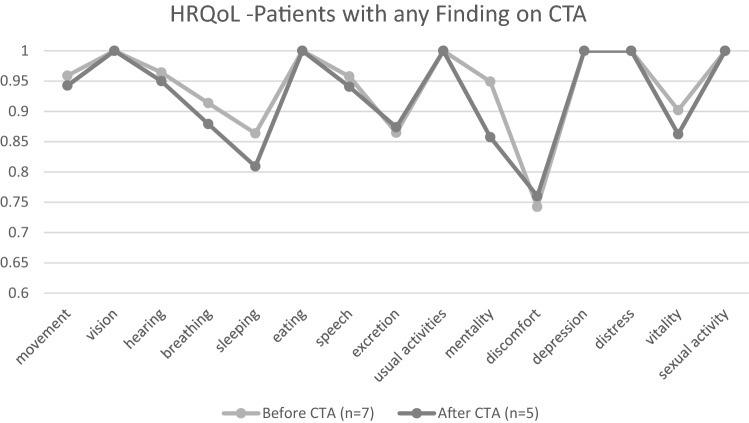
Health-related quality of life dimensions (15D score) of the participants prior to and after CTA with any findings (UIA, UIA suspicion or DAVF).

Out of seven participants who had cerebrovascular lesions in CTA, five returned the second HRQoL questionnaire. The UIA patient who was surgically treated experienced a large deterioration in the 15D score (> 0.035) prior to surgery, whereas the other UIA patients and the one with suspected dAVF experienced no change (Fig. 5).

### Screening costs

Screening and treatment costs have been listed in Table 2. We included all screening and treatment-related costs, even though only the pretreatment-related costs, such as CTA screens, letters, first clinical visits, travel costs, etc., were covered by the study funding, as screen-positive persons changed from being study participants to university hospital patients. The total screening costs were 18 817 EUR (22 627 USD), which translates to 3 763 EUR (4 524 USD) per screen-positive participant (n = 5). When summing together all the costs of active preventive treatment (microsurgical treatment) of one UIA patient as a result of screening, this amounts to 48 605 EUR (58 440 USD, screening, treatment and economic costs).

**Table 2:** Screening and treatment costs.

	Cost (EUR)	Valid for number of candidates (n or all)
Finnish Institute for Health and Welfare (research permit and consultation fee)	3 956.8	All
Postal costs	5.7 (399)	70
Lab costs	1.0 (45)	45
CT angiography (CTA)	250.0 (10 750)	43
DS angiography (DSA)	869.0 (1738)	2
Travelling costs	1232.50	all
Visit at the outpatient clinic	116.0 (696)	6
Treatment costs, neurosurgical ward (1 day)	654.0	1
Microsurgical operation	15 249.2	1
Inpatient day fee (2 days)	97.8	1
Treatment costs, neurosurgical ICU (24 h)	2 260.5	1
Sickness allowance (28 days)	1 726.5	1
Economic costs of sickness allowance^19^ (28 days)	350 (9800)	1
Altogether	48 605.3	All

All costs of UIA screening and active preventive treatment (microsurgical treatment) of one UIA patient. Economic costs have been estimated by using the evaluation of Confederation of Finnish Industries (EK) considering the costs of sickness absence^19^.

*CTA* Computed Tomography Angiography, *DSA* Digital Subtraction Angiography or Catheter Angiography, *ICU* Intensive Care Unit.

## Discussion

In this risk factor-based (age, gender and smoking) screening pilot for an asymptomatic but potentially life-threatening condition, five out of 43 female smokers (12%) were identified, each with one suspected UIA (4 diagnosed and one suspected but unconfirmed with DSA). A dural arteriovenous fistula was found in one study participant. The woman with the largest UIA (7 mm) was microsurgically treated without complications, and the patient with the dural arteriovenous fistula was scheduled for treatment. Four patients with small (2–3 mm) UIAs were advised to quit smoking, and all four were scheduled for a 1-year follow-up CTA. Two women with UIAs had hypertension, and one had a family history (≥ 1 first-degree members) of IAs. The proportion of hypertensive participants (n = 9, 21%) was in line with the general Finnish population; about one fifth of the population use antihypertensive medication^20^.

Previous data suggest that people with autosomal dominant polycystic kidney disease (ADPKD) have a significantly higher prevalence of UIAs^2^. In the largest study of screened ADPKD patients to date, UIAs (≥ 2 mm in size) were detected in 75 (9.2%) of the 812 ADPKD patients who underwent a magnetic resonance angiography (MRA) screening between 1989 and 2017 at the Mayo Clinic^21^. Even in ADPKD, selective or widespread screening for UIAs has remained questionable^21,22^, partly because ADPKD patients have a shortened life-expectancy due to the underlying kidney disease. In addition to ADPKD, UIAs may be found relatively frequently in families with a history of IAs. In the largest screening study of UIAs in first-degree relatives (parents, siblings or children) of SAH patients to date, UIAs were identified in 4.0% of 626 first-degree relatives (mean age 41 years; 52% women)^23^. Eighteen out of the 23 screen-positives underwent surgery (11 of which had UIAs smaller than 5 mm), which resulted in complications in 11 (disabling in one)^23^. In the largest UIA screening study of individuals having two or more first-degree relatives with a history of SAH, UIAs were identified in 11.1% of 458 relatives (median age 38 years; 58% women) in the first screening^24^. Of the identified UIAs, 25% were smaller than 2 mm and 51% were 3–5 mm in size^24^. No treatment results were reported^24^. In a multinational Familial Intracranial Aneurysm (FIA) Study, 548 subjects with a family history of ≥ 2 siblings (first-degree family members) or ≥ 3 any family members with IAs were screened with MRA, if they additionally had a history of ≥ 10 pack-years of smoking (or currently smoking) or hypertension^25^. The percentage of screen positives was 20.6%, but since 108 (96%) out of 113 found UIAs were small (< 7 mm in size), only 11 patients were treated surgically^25^. In comparison to the screening of individuals with ADPKD (a rare disease) and a family history of SAH, the population-wide effect of UIA screening in postmenopausal female smokers might be more significant, particularly since the number of 50 to 60-year-old female smokers is substantial in many countries. For example, in 2018, 16.3% of women aged 45 to 64 years old were current cigarette smokers in the United States^26^, whereas the estimated prevalence of ADPKD in the Unites States is 0.04%^27^. This idea is supported by the results of a nested case–control study, which reported an UIA prevalence of 19% in female smokers who had undergone a brain magnetic resonance angiography for a reason unrelated to intracranial aneurysms^28^.

Preventive health screenings have the fundamental goal of detecting potential health concerns early, and this should help individuals to stay healthy. Whether the shared information about the high risk of SAH has resulted in a long-term smoking cessation among the screened women needs to be followed up and studied. In addition to the possibility of reducing smoking rates in screened individuals, the targeted screening should reduce SAH-related morbidity and mortality. In this respect, familial UIA screenings may not reduce SAH-related morbidity and mortality. In the largest familial SAH study to date, 156 (3.0%) out of 5282 SAH patients had one first-degree family member with a history of SAH^29^. In one of the largest twin studies conducted to date, study authors identified 6 (1.2%) concordant (5 monozygotic and 1 opposite sex) and 492 discordant twin pairs for SAH out of a total of 504 SAH cases, suggesting that familial SAHs are rare, and that the relative contribution of inherited genes to SAH is moderate at most^30^. Contrary to the low familial SAH risk, the risk of SAH is vastly increased in hypertensive smokers. In fact, the incidence of SAH is over 14 times higher (171 per 100,000 person-years) in women with a history of active smoking and high blood pressure (systolic blood pressure ≥ 159 mmHg) than in normotensive women without a smoking history^31^. If it is accurate that close to 50% of smoking women with diagnosed UIAs suffer from SAH at some point in their lives^10^, and that the incidence of SAH in female smokers is exceptional^31^, a targeted UIA screening of female smokers (both normotensive and hypertensive) could perhaps be more effective than the familial screening. This might be especially true if the screening identifies women with small UIAs, thus enabling non-invasive, i.e. complication free interventions, such as smoking cessation. Smoking cessation decreases the risk of SAH, and five years after quitting, the risk of SAH among former smokers appears to be close to that of people who have never smoked^8^.

It remains speculative whether screening for UIAs in female smokers would be cost-effective, and could increase the quality of life. In our study, the screening costs were 3 763 EUR (4 524 USD) per screen-positive woman. If estimated that the operated 7 mm UIA had ruptured at some point in life^10^, and if all screening and treatment-related costs were allocated to the treated screen-positive smoker, the prevention of one aSAH amounted to 48 605 EUR (58 440 USD). Those participants that returned the HRQOL questionnaires before and after the CTA imaging did not deteriorate in the HRQOL. However, one screen-positive woman reported a decline in her HRQOL score after the UIA diagnosis.

Our pilot has a few strengths. Since the overall prevalence of UIAs^2^ and incidence of SAH^7^ in Finland do not differ from other countries also reporting reliable prevalence and incidence estimates, UIAs may also be found at a similar frequency in 50 to 60-year-old female smokers in other countries. We chose to screen people with CTA alone, thus decreasing the likelihood of finding false positives and incidental findings, which are more common in MR imaging studies. Most importantly, we tried to diminish the risk of a recruitment bias by inviting only true current smokers. Our study also has limitations. The response rate for the preliminary invitation letters was not high, which may have indeed led to a recruitment bias. Furthermore, the postal strike during 11.–27.11.2019 and the coronavirus pandemic-related restrictions in recruiting research patients between 17.3.–29.7.2020 and 23.11.–13.12.2020 prolonged the study protocol, and may have affected the recruitment protocol. In addition, we cannot be certain that by selecting high-risk individuals for screening, we avoided the most serious harm of screenings, i.e. overdiagnosis. Finally, the number of screened women in our pilot is small. However, without a pilot and a subsequent international peer review, which estimates the scientific justification of the suggested approach, it is challenging to obtain a research permission for larger screening studies from hospital ethics committees, which follow the Declaration of Helsinki as a statement of ethical principles for medical research involving human subjects.

## Conclusions

In conclusion, our findings suggest that the prevalence of UIA is above 10% in 50 to 60-year-old smoking women and that these UIAs can be detected at the point when they are still small. Our pilot study results may warrant a larger and multinational screening study of 50 to 60-year-old female smokers.

## Supplementary Information

Supplementary Information.

## Data Availability

Pseudonymized research data are personal data according to Finnish legislation and cannot be shared outside the Helsinki University Hospital.
