# Supplementary material for: Screening of unruptured intracranial aneurysms in 50 to 60-year-old female smokers: a pilot study
Source: Sci Rep. 2021 Dec 9;11:23729. doi: 10.1038/s41598-021-02963-z (PMC8660906; doi:10.1038/s41598-021-02963-z)
Supplement: Supplementary file 1 — Supplementary Information. [file 41598_2021_2963_MOESM1_ESM.pdf]

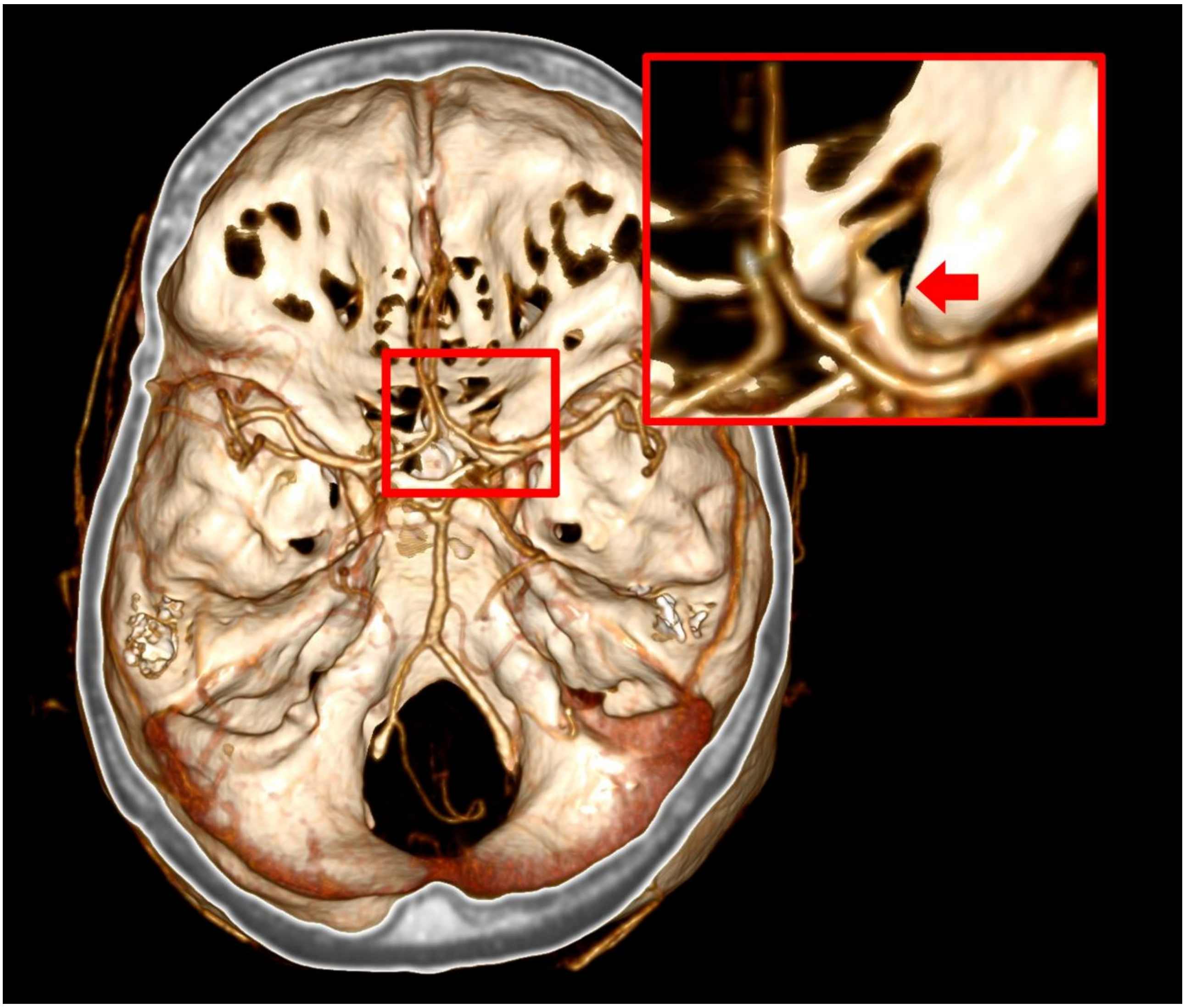

Supplementary Figure. One suspected UIA of the right internal carotid artery-ophtalmic segment has not been confirmed due to DSA cancellations by the patient.
